# Osur et al.'s *Implementation of misoprostol for postabortion care in Kenya and Uganda: a qualitative evaluation*

**DOI:** 10.3402/gha.v6i0.21786

**Published:** 2013-07-18

**Authors:** Molly Moran, Joanna Ortega, Nuriye Nalan-Sahin Hodoglugil

**Affiliations:** 1 Venture Strategies Innovations, Irvine, CA, USA; 2 Venture Strategies Innovations, Berkeley, CA, USA

Osur and colleagues’ article on the evaluation findings of implementing misoprostol for postabortion care (PAC) in Kenya and Uganda provides valuable lessons learned and best practices. Venture Strategies Innovations (VSI), a global health non-profit organization, is currently introducing misoprostol for PAC with the Ministries of Health (MOH) in Rwanda and Zimbabwe, and based on our ongoing implementation experiences, we would suggest a few additional lessons.

First, Osur and colleagues rightly identify that the creation of an enabling environment is one of the most vital elements of successfully piloting misoprostol for PAC. In our experience, building broad consensus around the need for misoprostol among key stakeholders, including NGO partners, departments within the MOH, and multilateral organizations working in maternal health, is critical, and should be embarked upon prior to the pilot and continuously strengthened to ensure a sustainable scale-up.

Second, Osur and colleagues highlight the importance of creating a plan to maintain misoprostol supplies throughout initial implementation and also note that they had to register the drug in Kenya before beginning implementation. Our experience has been that registration has not been a prerequisite to pilot implementation, as strong MOH support has allowed for tablet donations prior to drug registration. Additionally, we have found that collaborative strategizing for long-term commodity security as well as registering more than one misoprostol product to ensure supply-side availability should accompany planning for tablet procurement during initial implementation. Alongside this, institutionalization activities such as including misoprostol for PAC in health professionals’ in-service training curricula and creating MOH-endorsed tools such as misoprostol dosage pocket guides should be undertaken.

Third, Osur and colleagues note that women's perception of misoprostol as compared to other treatment options can impact acceptance and uptake of the drug. This finding is corroborated by qualitative data from Zimbabwe, where providers have reported that some women prefer dilatation and curettage (D&C) because of the belief that it will be more effective than misoprostol in ‘cleansing’ the remaining products of conception. In Shona, one of the principal languages in Zimbabwe, D&C is translated as ‘cleansing of the womb’. These findings highlight the need for culturally appropriate Information, Education and Communication (IEC) materials that providers can use to explain to women the benefits of using misoprostol for PAC, which include that the drug is non-surgical, safe (1, 2), has comparable efficacy rates to manual vacuum aspiration (MVA) (3, 4), and, in most countries (including Zimbabwe and Rwanda), is less expensive than other treatment options.

Finally, our experiences have taught us that in an initial implementation it is important to introduce misoprostol universally in a wide range of facility levels, and in facilities that do and do not offer MVA as a treatment option. This enables monitoring of variations in misoprostol uptake across facility levels and can highlight implementation challenges specific to either initiating misoprostol for PAC within an existing referral system or integrating misoprostol into existing PAC services. In addition, it can unveil important provider preferences for treatment options. This varies from the sampling design utilized by Osur et al., where facilities were purposefully selected based on demand for PAC, willingness of providers and administrators to support implementation of the pilot program, and availability of MVA. Examples of the lessons that we have learned from monitoring misoprostol uptake across a wide range of facilities come from a comparison of implementation in Rwanda and Zimbabwe. As soon as misoprostol was introduced in Rwanda, providers at health centers and hospitals began using it to treat the majority of incomplete abortion and miscarriage cases (90% in the first month of implementation) (5). However, in Zimbabwe, while misoprostol uptake at health centers has also been immediate, uptake at hospitals has been low in the initial project months because of provider preference for D&C, which is possibly attributable to financial incentives and perceived efficiency. Understanding the localized factors that lead to differences in misoprostol uptake will allow for the creation of a more informed scale-up plan that can lead to rapid expansion of PAC services.

*Molly Moran* Venture Strategies Innovations, Irvine, CA, USA Email: mmoran@vsinnovations.org*Joanna Ortega* Venture Strategies Innovations, Berkeley, CA, USA*Nuriye Nalan-Sahin Hodoglugil* Venture Strategies Innovations, Berkeley, CA, USA
